# Interface‐Enhanced and Self‐Guided Growth of 2D Interlayer Heterostructure Wafers with Vertically Aligned Van Der Waals Layers

**DOI:** 10.1002/advs.202412690

**Published:** 2025-02-17

**Authors:** Yi Hu, Xingli Wang, Xingguo Wang, Yue Gong, Zikun Tang, Guangchao Zhao, Weng Hou Yip, Jingyi Liu, Seoung Bum Lim, Mohamed Boutchich, Philippe Coquet, Shu Ping Lau, Beng Kang Tay

**Affiliations:** ^1^ Centre for Micro‐ and Nano‐Electronics (CMNE) School of Electrical and Electronic Engineering Nanyang Technological University Singapore 638798 Singapore; ^2^ CINTRA IRL 3288 (CNRS NTU THALES) Nanyang Technological University Singapore 637553 Singapore; ^3^ Department of Applied Physics Hong Kong Polytechnic University Hung Hom Kowloon Hong Kong 999077 P. R. China; ^4^ Interdisciplinary Graduate School Nanyang Technological University Singapore 639798 Singapore; ^5^ Sorbonne Université CNRS Laboratoire de Génie Electrique et Electronique de Paris Paris 75252 France; ^6^ Institut d'Electronique Microélectronique et Nanotechnologie (IEMN) CNRS Université de Lille Lille 60069 France

**Keywords:** interface‐enhanced selenization, rectification effect, self‐guided growth, Si‐compatible integration, wafer‐scale 2D heterostructure

## Abstract

2D heterostructures have garnered significant interest in the scientific community owing to their exceptional carrier transport properties and tunable band alignment. The fabrication of these heterostructures on a wafer scale is crucial for advancing industrial applications but remains particularly challenging for metals with low sulfidation activity, such as Hf. Herein, the one‐pot method is developed for fabricating wafer‐scale HfSe_2_/WSe_2_ interlayer heterostructures with vertically aligned van der Waals layers via interface‐enhanced selenization and self‐guided growth. By depositing a W layer (high sulfidation activity) over a Hf layer, followed by a one‐pot selenization process, the chemical combination between Hf and Se atoms is enhanced through interfacial Se diffusion and confined lattice reaction. Moreover, the WSe_2_ layers grow perpendicular to the substrate and further guide the crystallization of the bottom HfSe_2_ layers. The resulting heterostructures, characterized by covalent bonds, demonstrate significant charge transfer, enhanced piezoelectricity, notable rectification effects, and Si‐compatible transistor integration. This interface‐enhanced selenization and self‐guided growth pathway may provide valuable insights into the fabrication of covalently connected interlayer heterostructures involving metals with low sulfidation activity, as well as the development of high‐density integrated circuits.

## Graphical abstract

Through the one‐pot interface‐enhanced selenization and self‐guided growth method, wafer‐scale HfSe_2_/WSe_2_ interlayer heterostructures with vertically aligned van der Waals layers are fabricated. The resulting heterostructures, characterized by covalent bonds, demonstrate significant charge transfer, enhanced piezoelectricity, notable rectification effects, and Si‐compatible transistor integration.

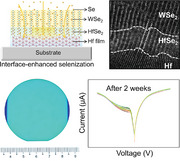

## 1 Introduction

2D interlayer and intralayer heterostructures, artificially reassembled by isolated 2D layers, offer an ideal platform to study novel fundamental physics and electronics.^[^ ^1^, ^2^, ^3^, ^4^, ^5^, ^6^, ^7^, ^8^, ^9^, ^10^, ^11^, ^12^, ^13^, ^14^, ^15^ ^]^ These 2D heterostructures provide greater flexibility and complexity in assembly compared with traditional crystals, demonstrating significant synergetic effects including charge redistribution and structural evolution. These characteristics enable the engineering of novel entities, such as excitons,^[^ ^13^, ^14^, ^15^, ^16^ ^]^ electron quantum metamaterials,^[^ ^17^, ^18^ ^]^ spin and valley electronics,^[^ ^19^, ^20^, ^21^ ^]^ and Moiré superlattices.^[^ ^16^, ^22^, ^23^ ^]^ Especially, 2D heterostructures allow diverse combinations and precise control of the band energy level alignment, rendering them suitable for high‐performance, low‐power devices, such as p‐n junctions,^[^ ^5^ ^]^ light‐emitting diodes,^[^ ^7^ ^]^ metal‐semiconductor contacting transistors,^[^ ^8^, ^9^ ^]^ and tunneling diodes.^[^ ^24^, ^25^ ^]^ State‐of‐the‐art technologies for fabricating wafer‐scale 2D heterostructures include chemical vapor deposition (CVD),^[^ ^26^, ^27^ ^]^ pulsed laser deposition,^[^ ^28^ ^]^ and sulfurization/selenization of deposited metal films,^[^ ^29^, ^30^ ^]^ or methods such as artificially stacking 2D layers using programmed vacuum stacking^[^ ^31^ ^]^ and polymer‐assisted wet transfer.^[^ ^32^ ^]^ Those methods effectively synthesize interlayer 2D heterostructure but remain challenging in fabricating intralayer 2D heterostructures or interlayer heterostructures with covalently bonded van der Waals (vdW) layers. Additionally, substrate‐parallel intralayer 2D heterostructures are inefficient in terms of maximizing the wafer surface area usage, which hinders high‐density transistor integration.

**Figure 1:**
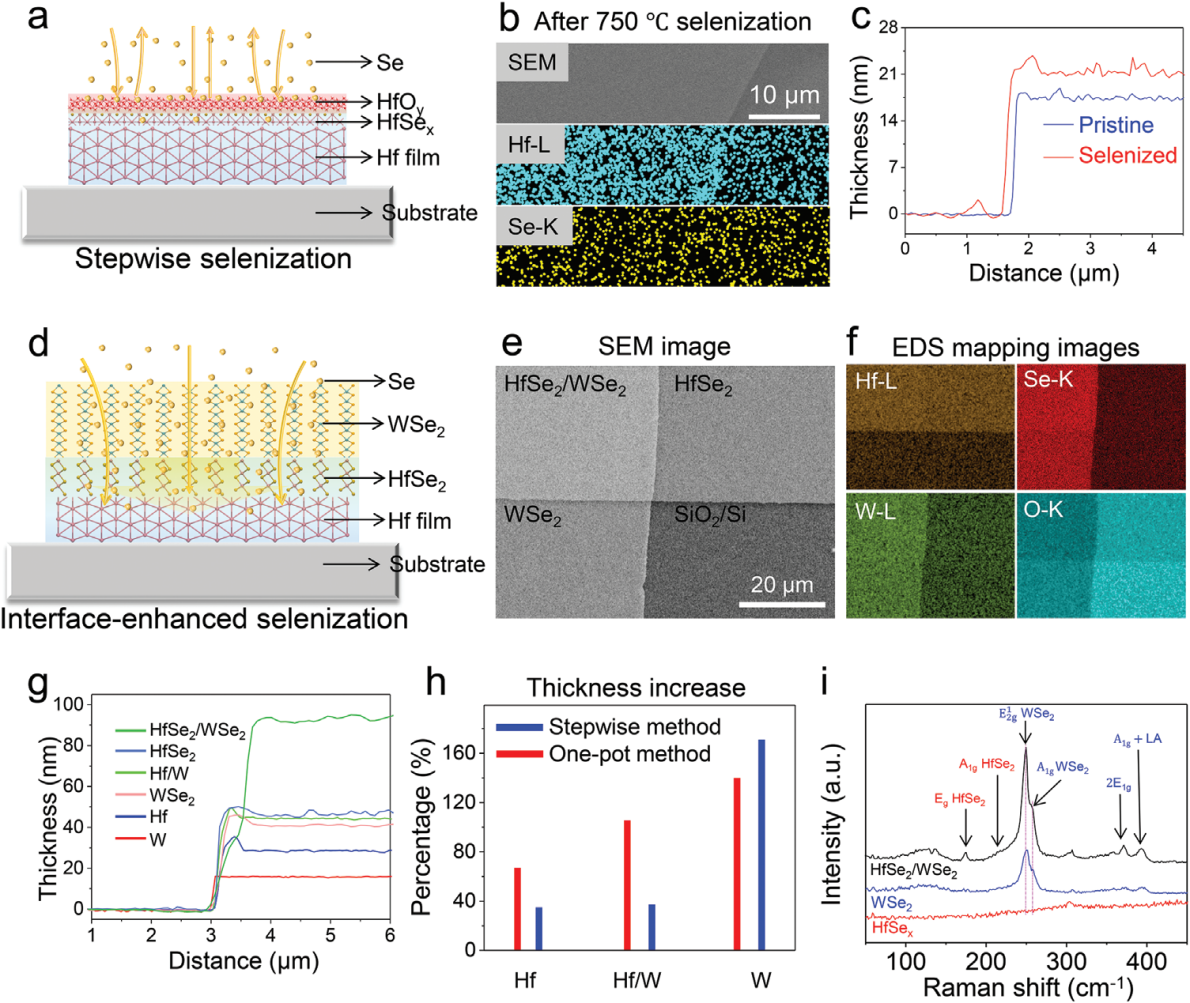
Comparison of the stepwise method and one‐pot interface‐enhanced selenization method for preparing HfSe_2_/WSe_2_ heterostructures. a) Schematic illustration of the selenization of pure Hf metal film using the stepwise method. b) SEM and EDS mapping images of the Hf film on a SiO_2_/Si substrate after selenization. c) AFM height profiles of pristine Hf film (blue line) and the film after selenization at 750 °C (red line). d) Schematic illustration of the selenization of Hf/W metal films using a one‐pot interface‐enhanced method. e,f) SEM image and corresponding EDS mapping images of Hf/W, W and Hf films after one‐pot interface‐enhanced selenization at 750 °C. g) Height profiles of Hf/W, W and Hf films before and after one‐pot interface‐enhanced selenization at 750 °C. h) Percentage increase in the thickness of Hf, W/Hf, and W films after selenization through the stepwise and one‐pot methods. i) Raman spectra of the pure Hf film, pure W film, and Hf/W heterogenous film after selenization.

Recently, direct sulfurization/selenization of deposited metal films has shown promise for fabricating uniform, multilayered stacked vdW heterostructures on a wafer scale.^[^ ^29^, ^30^, ^33^ ^]^ However, for metals with low reactivity and high sulfurization/selenization reaction energy barriers, it remains challenging to directly prepare uniform wafer‐scale 2D heterostructures with a high‐quality interface to ensure considerable interlayer coupling or charge transfer. Hf, in particular, is a typical inactive metal with a high density and a stable oxide surface layer.^[^ ^34^ ^]^ Moreover, Hf‐related compounds are critical for modern semiconductor devices. For example, HfSe_2_ is a layered 2D semiconductor with Si‐equivalent band gaps ranging from 0.9 to 1.2 eV (bulk to monolayer), and HfO_2_ is comparable to the technologically desirable high‐κ dielectrics.^[^ ^35^ ^]^ As HfSe_2_ exhibits considerable electron affinity energy (5.2 eV) and work function (5.5 eV), it is a high‐potential component for constructing tunneling heterostructures with conventional type‐II band alignment and gate‐controlled band alignment transition from type‐II to type‐III.^[^ ^36^, ^37^ ^]^ However, owing to its low reactivity, pure Hf metal films are difficult to sulfurize or selenize in a facile manner, usually leading to limited increase of film thickness after selenization and surface contamination during stepwise metal film deposition and selenization (**Figure** **1**a–c). To date, the few reported preparations of hafnium chalcogenides have used hafnium chlorides as precursors, which is unconducive to the fabrication of wafer‐scale heterostructures with clean interfaces.

Thus, in this study, we enhanced the selenization of Hf metal films and guided the growth of 2D interlayer heterostructures with vdW layers aligned perpendicular to the substrate through the assistance of top W metal layers. The successful preparation of HfSe_2_/WSe_2_ interlayer heterostructures was verified through an increase in thickness, transmission electron microscopy (TEM), energy dispersive X‐ray spectroscopy (EDS) mapping, Raman spectrum, X‐ray diffraction (XRD) patterns, and X‐ray photoelectron spectroscopy (XPS) analyses. The thickness increases of the W/Hf films produced by interface‐enhanced selenization were three times larger than that achieved through stepwise selenization, confirming a higher degree of selenization of Hf metal films. High‐resolution cross‐sectional TEM results revealed that the growth of HfSe_2_ was guided by both the top WSe_2_ layer and bottom Hf metal film, resulting in an interlayer HfSe_2_/WSe_2_ heterostructure aligned perpendicularly to the substrate surface. Furthermore, a 2‐inch wafer of the HfSe_2_/WSe_2_ interlayer heterostructure was demonstrated, showcasing excellent flatness and uniformity and highlighting its potential for industrial applications. Benefiting from the high‐quality, covalently linked interfaces, the 2D heterostructures exhibited significant charge transfer and enhanced piezoelectricity. The smooth band alignment resulted in notable electrical rectification behaviors with a rectification ratio of 12.5. By integrating the n‐Si substrate with the HfSe_2_ and WSe_2_ layers, the junctions suggested tunnel breakdown at 4 V and minimal performance variation even after 50 scan cycles. Lastly, the electrical performance of the 24 device groups remained highly stable and repeatable, even after two weeks of exposure to ambient air.

## 2 Results

### 2.1 Interface‐Enhanced Selenization of Hf Metal Film

As shown in Figure S1a (Supporting Information) and Figure 1a, the stepwise method involves sequential deposition and selenization of metal films by using magnetron sputtering and CVD. Initially, a Hf metal film is slowly deposited onto a Si substrate via magnetron sputtering. The Hf metal film is then placed into a single‐zone tube furnace for selenization at 750 °C, producing a partially selenized Hf film (HfSe_x_). Subsequently, W metal film deposition and CVD selenization are carried out, and a WSe_2_ layer is formed on top of the HfSe_x_ film. The color of the Hf film changes after selenization, and the surface remains uniform without noticeable particles (Figure S2a,b, Supporting Information). However, EDS mapping images of selenized Hf film show negligible Se signals in the Hf film (Figure 1b). As Hf is a highly dense metal with low reactivity, and owing to air exposure, it readily oxidizes to form an inert hafnium oxide film on the surface.^[^ ^34^ ^]^ Moreover, Se vapors tend to desorb from the Hf surface in an open environment (Figure 1a). As a result, the Hf film is insufficiently selenized, as demonstrated by the limited thickness increase of 6.3 nm after selenization at 750 °C from the initial 17.2‐nm‐thick hafnium film. This is much less than the thickness increase after the formation of HfSe_2_ (Figure 1c; Figure S2c, Supporting Information). Therefore, the actual thickness of HfSe_x_ is less than 6.3 nm, suggesting weak chemical interactions between Hf and Se. Additionally, owing to the exposure of Hf film to the air environment during the second W metal film deposition, the HfSe_x_ film undergoes surface contamination, which reduces the interfacial coupling of the heterostructure and potentially leads to charge carrier trapping (Figure S3a,b, Supporting Information). In contrast, the thickness of the second W film increases significantly from 18.6 to 50.5 nm after selenization, indicating nearly complete selenization of the W film and the formation of WSe_2_ vdW layers (Figure S3c,d, Supporting Information). Even after the second selenization, the thickness of the HfSe_x_ film remains nearly unchanged, and the increased thickness of HfSe_x_/WSe_2_ is attributable entirely to the thickness increase during W selenization. The corresponding EDS mapping images indicate constant Se signal intensity in the Hf/W and pure W films, confirming that only the W metal film undergoes selenization (Figure S4, Supporting Information). These preliminary experimental results indicate that the stepwise metal deposition and selenization method for preparing 2D heterostructures is undesirable for inert metals like Hf, which readily form a dense surface oxide layer.

To address this, we propose a one‐pot method to enhance the selenization of Hf/W metal films through interface‐accelerated Se diffusion and reaction (Figure 1d; Figure S1b, Supporting Information). This method offers three main advantages: a clean interface, the absence of a Hf oxidation layer, and interface‐enhanced selenization. As the sequential deposition of Hf and W metal films is performed in a vacuum environment, the heterostructure interface remains free from contamination by dust and molecules. During selenization, the high reactivity of W with Se leads to the formation of WSe_2_, which facilitates the capture of Se atoms. Owing to the difference in the concentration of Se atoms at the interface, the WSe_2_ layer facilitates the diffusion of Se atoms into the Hf film. The diffused Se atoms have a short interaction distance with the Hf atoms confined in the lattice space, increasing the probability of atom collisions and leading to the formation of HfSe_2_. Consequently, the thickness of the Hf/W film nearly doubles owing to this interface‐enhanced selenization process (Figure 1g; Figure S5a,b, Supporting Information). Particularly, adjacent pure Hf films also exhibit significant thickness increases, from 28.2 to 47.2 nm, suggesting that the Se atoms migrate laterally through the HfSe_2_ lattice to the adjacent pure Hf films. The calculated thickness increase contributed by the selenization of Hf in the Hf/W metal film is ≈24.3 nm, which exceeds the thickness increase of a single Hf film after selenization (18.9 nm).

Optical images of the Hf/W, W, and Hf films show consistent uniformity and color variations associated with the thickness increase (Figure S5c,d, Supporting Information). EDS mapping of Se reveals detectable signals in the pure Hf film and higher Se intensity in the Hf/W film compared with the pure W film (Figure 1e,f). These results indicate that the top W film enhances the selenization of the underlying Hf and extends to the side Hf metal films. The percentage increase in the thickness of Hf, W, and W/Hf films selenized using either the stepwise or one‐pot methods more clearly illustrates the selenization degree (Figure 1h). The thickness increase of Hf selenized using the one‐pot method (67%) is nearly two times that associated with the stepwise method (35%). Similarly, the thickness increase of Hf/W selenized using the one‐pot method (105%) is three times larger compared to the stepwise method (37%). This enhancement suggests a higher degree of selenization and superior crystal quality of the metal selenide films. Raman spectra of the three Hf, Hf/W, and W films after selenization are shown in Figure 1i. Although characteristic Raman peaks of WSe_2_ are detected in the selenized pure W metal film, no peaks of HfSe_2_ are detected in the directly selenized Hf film, indicating that the W film is more readily selenized than the inert Hf film. However, peaks of both WSe_2_ and HfSe_2_ are detected in the Hf/W films after interface‐enhanced selenization (black line in Figure 1i). The WSe_2_ and HfSe_2_ peaks exhibit red and blue shifts, respectively, confirming strong coupling within the heterostructure.^[^ ^38^ ^]^

### 2.2 Guided Growth of HfSe_2_/WSe_2_ Interlayer Heterostructure

**Figure 2:**
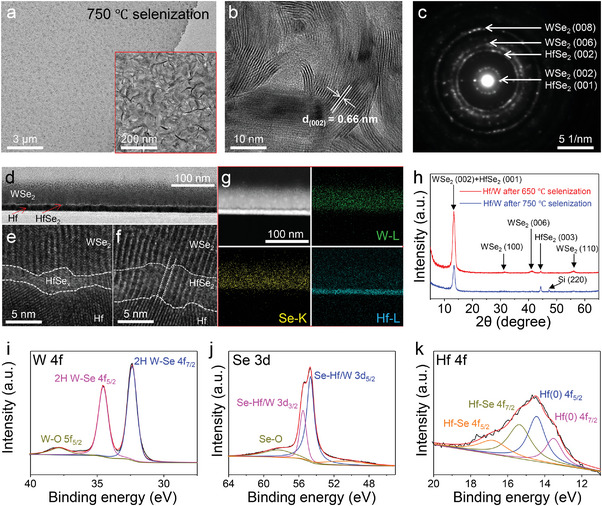
Crystalline structures and chemical compositions of the HfSe_2_/WSe_2_ interlayer heterostructure prepared via one‐pot selenization method. a) Low‐magnified TEM image of the HfSe_2_/WSe_2_ heterostructure along the top‐view. The inset shows a magnified TEM image. b,c) High‐resolution TEM image and corresponding SAED patterns of the HfSe_2_/WSe_2_ heterostructure. d) Cross‐sectional low‐resolution TEM image of the HfSe_2_/WSe_2_ interlayer heterostructure prepared by one‐pot selenization at 700 °C. e,f) High‐resolution TEM image of HfSe_2_‐WSe_2_ interface with different degrees of selenization. g) TEM images and corresponding EDS elemental mapping images of HfSe_2_/WSe_2_ interlayer heterostructure. h) XRD patterns of HfSe_2_/WSe_2_ heterostructure prepared by selenizing the Hf/W film at 650 and 750 °C. i–k) XPS core level spectra of (i) W 4f, (j) Se 3d, and (k) Hf 4f in the HfSe_2_/WSe_2_ heterostructure.

To reveal the crystal quality and lattice structure of the HfSe_2_/WSe_2_ heterostructure, TEM characterizations were collected through top and cross‐section views (**Figure** **2**). The HfSe_2_/WSe_2_ heterostructure films can be completely transferred from Si substrate to Cu copper grids and show homogeneous TEM contrast and element distributions (Figure 2a; Figure S6, Supporting Information), confirming its high uniformity and compact structure. High‐resolution TEM image (HRTEM) shows regular lattice stripes with large distances (Figure 2b). Corresponding SAED also presents circular diffraction patterns, indicating typical polycrystalline features of the HfSe_2_/WSe_2_ heterostructure (Figure 2c). The calibrated lattice indexes demonstrate the exposure of the (0 0 n) lattice plane, suggesting the vertically aligned vdW layers of the interlayer HfSe_2_/WSe_2_ heterostructure. Moreover, Cross‐sectional TEM analysis is performed after carbon deposition and focused ion beam (FIB) cutting to confirm the interface quality and growth behavior. The cross‐section of the as‐prepared HfSe_2_/WSe_2_ heterostructure reveals an apparent three‐layer structure with different contrasts, which can be assigned to the WSe_2_ layer, HfSe_2_ layer, and Hf film from top to bottom (Figure 2d). The selenization of this HfSe_2_/WSe_2_ heterostructure is performed at a low temperature of 700 °C to maintain well‐defined boundaries between the layers and reveal the selenization process. Thus, a thin layer of Hf is successfully selenized, resulting in a HfSe_2_ layer with a thickness of less than 5 nm. The selenization process and guided growth are revealed by HRTEM images at the HfSe_2_/WSe_2_ interface (Figure 2e,f). First, WSe_2_ is formed, guided by the bottom Hf metal film, resulting in vdW layers aligned perpendicular to the substrate surface (Figure 2e). The surface of the Hf film is initially selenized at this stage, showing mixed and complex lattice stripes. With the increased degree of selenization of the Hf film, the thickness of HfSe_2_ increases, resulting in more regular vdW lattice stripes (Figure 2f). Especially, the vdW interplanar spacing values (0 0 n) in both WSe_2_ and HfSe_2_ are nearly identical and correspond to three times the Hf lattice spacing, indicating guided growth of the HfSe_2_ layers by the top WSe_2_ and Hf layers (white lines in Figure 2f). The corresponding EDS mapping confirms that the W, Se, and Hf signals are consistent with the chemical composition of the heterogeneous films (Figure 2g). However, owing to the ultrathin feature of the HfSe_2_ layer, it is challenging to distinguish directly from the bottom Hf and top WSe_2_ layers through EDS mapping. The high‐resolution lattice characterizations, combined with EDS mapping, confirm the formation of HfSe_2_ and the self‐guided growth of interlayer heterostructures with the vdW layer aligned perpendicular to the substrate.

XRD patterns and XPS spectra are assessed to reveal the heterostructure's chemical composition, interlayer coupling, and crystal orientation. The XRD patterns of Hf/W films selenized at 650 and 750 °C show typical (0 0 n) lattice planes of WSe_2_ and HfSe_2_ (Figure 2h). As the selenization temperature increases from 650 to 750 °C, the crystal domain size expands, and the crystal quality improves, as evidenced by sharper XRD peaks and the disappearance of the (1 0 0) and (1 1 0) peaks of WSe_2_. However, both the Raman peaks and XRD peaks of HfSe_2_ are weak, likely because of their ultrathin thickness and the shielding effect of the top WSe_2_ layer. The XPS spectra are obtained to assess the chemical environments of W, Hf, and Se (Figure 2i–k). The W 4f core orbit spectrum exhibits two strong peaks at 34.5 and 32.4 eV, corresponding to the binding energies of the 4f_5/2_ and 4f_7/2_ line peaks of 2H WSe_2_, respectively. The 3d_5/2_ and 3d_3/2_ doublet at 54.6 and 55.6 eV in the Se 3d core level spectrum also confirms the successful selenization reaction between Se and the Hf/W metal films. In contrast, the Hf 4f core orbit spectra indicate partial selenization of Hf, as confirmed by the co‐existence of Hf–Se lines and zero‐valence Hf lines (Figure 2k). However, the XPS spectra for both W and Se display oxidation states, while no hafnium oxide peak is observed, despite Hf being more prone to oxidation. The oxidation of WSe_2_ can be ascribed to the surface air exposure, suggesting the critical role of the top W layer in accelerating the selenization of Hf film and preventing the oxidation of HfSe_2_.

### 2.3 Wafer‐Scale Preparation of the HfSe_2_/WSe_2_ Interlayer Heterostructures

**Figure 3:**
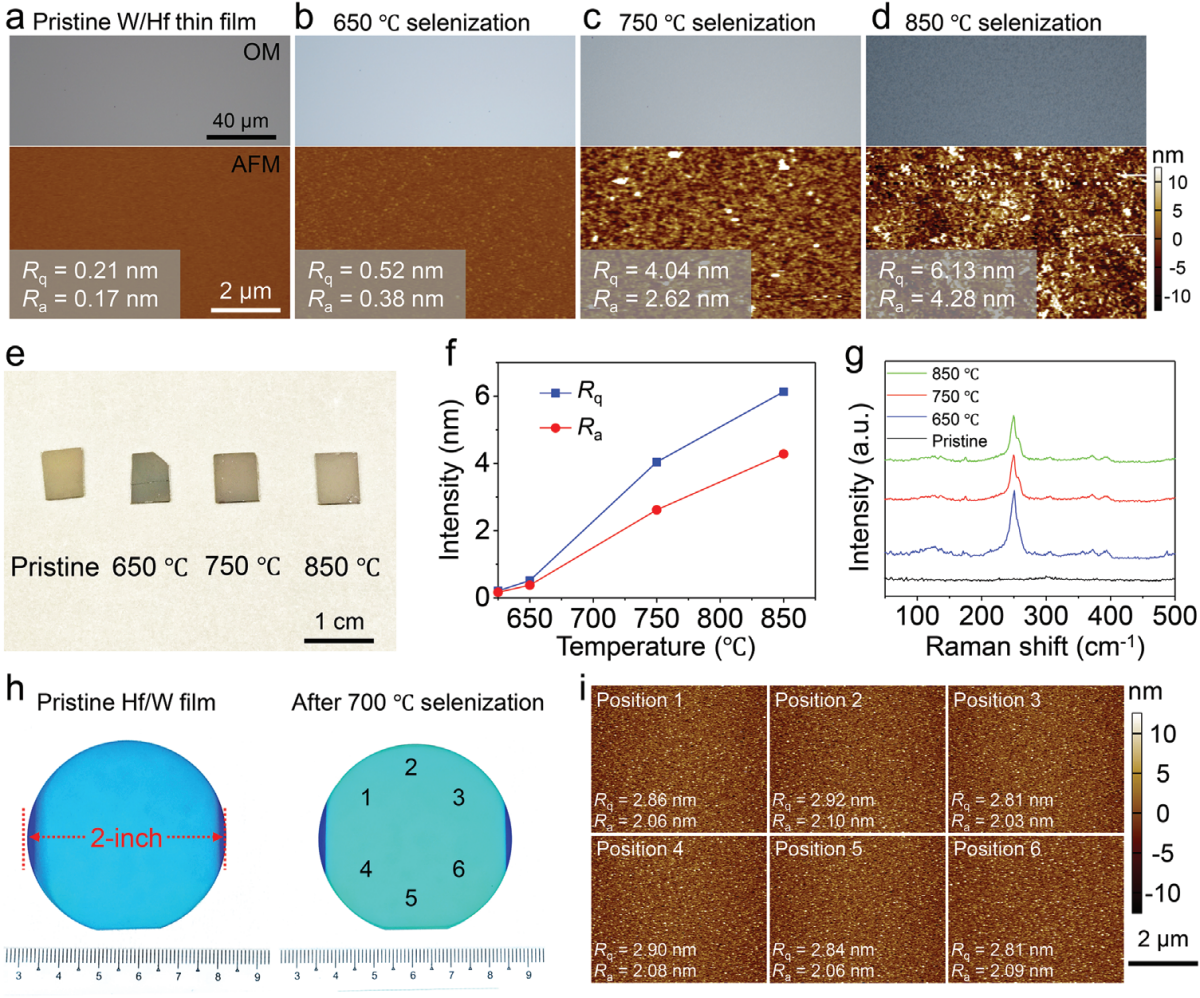
Wafer‐scale preparation of the interlayer HfSe_2_/WSe_2_ heterostructure. a–d) Optical images and AFM images of Hf/W films on a Si substrate before and after 650, 750, and 850 °C selenization. The corresponding values of *R*_a_ and *R*_q_ values are specified in the left corner of the images. e) Digital photos of the Hf/W films on a Si substrate before and after 650, 750, and 850 °C selenization. f) *R*_q_ and *R*_a_ intensities of the films on Si substrate as a function of growth temperature. g) Raman spectra of Hf/W films on a Si substrate before and after 650, 750, and 850 °C selenization. h) Digital photos of 2‐inch Hf/W metal film wafer before and after 700 °C selenization. i) AFM images collected at six positions on the HfSe_2_/WSe_2_ heterostructure marked in (h).

The XRD patterns highlight the importance of temperature in controlling the domain size of the heterostructure. To explore this aspect, Hf/W films are selenized at 650, 750, and 850 °C, and the morphology and surface roughness are evaluated using AFM and optical microscope (**Figure** **3**). The pristine Hf/W metal film is ultra‐flat, with the values of arithmetic average height (*R*_a_) and root mean square deviation of the roughness (*R*_q_) being 0.17 and 0.21 nm, respectively (Figure 3a). After selenization, the color of the optical image of the Hf/W film on the Si substrate changes from dark gray to light gray (650 °C) and then back to dark gray (750 °C) (Figure 3a–c,e), indicating changes in composition and optical band gap. The Hf/W metal films selenized at 650 and 750 °C show a clean and smooth surface (Figure 3b,c). The roughness parameters (*R*_a_/*R*_q_) of the Hf/W films increase to 0.38/0.52 nm (650 °C) and 2.62/4.04 nm (750 °C). However, after selenization at 850 °C, the optical image reveals a prominent granular surface, and the dark field optical image shows obvious optical nonuniformity (Figure 3d; Figure S7a, Supporting Information). The corresponding AFM image demonstrates concave pores and flake‐like surfaces, resulting in higher roughness values of *R*_a_ = 6.13 nm and *R*_q_ = 4.28 (Figure 3d). Surface cracks in the heterostructure films become more pronounced at higher selenization temperatures of 900 and 950 °C (Figure S7b–f, Supporting Information). Film degradation and surface particles can be easily observed even in bright‐field optical images and digital photos, indicating the migration and convergence of W and Hf atoms at high temperatures (Figure S7, Supporting Information). Plotting the roughness parameters (*R*_a_ and *R*_q_) against the selenization temperature reveals significant positive linear correlations, indicating that the selenization degree and quality of the heterostructure can be effectively controlled (Figure 3f). Compared with selenization at 650 °C, the Hf/W metal films prepared at 750 and 850 °C exhibit stronger and sharper HfSe_2_ Raman peaks but comparable WSe_2_ Raman signals (Figure 3g). Therefore, the analysis of the uniformity and crystal quality of heterostructures prepared at different temperatures suggests that heterostructures with excellent electrical performance can be achieved through selenization at ≈700 °C, ensuring high uniformity and strong interlayer coupling.

Furthermore, a 2‐inch heterostructure wafer is demonstrated on a SiO_2_/Si substrate (Figure 3h). The color changes in the digital images confirm the successful selenization of the metal films. The optical images collected at six points on a 2‐inch HfSe_2_/WSe_2_ heterostructure wafer show identical contrasting colors, indicating a constant thickness across the wafer (Figure S8a–f, Supporting Information). Notably, the surface of the Hf/W films exhibits excellent flatness and smoothness both before and after 700 °C selenization. The surface roughness (*R*_a_ and *R*_q_) parameters recorded at six positions on the 2‐inch HfSe_2_/WSe_2_ heterostructure wafer exhibit minimal variations (Figure 3i; Figure S8g, Supporting Information), confirming the high uniformity of the HfSe_2_/WSe_2_ interlayer heterostructure wafer and highlighting its potential for large‐scale, high‐intensity integrated circuits. Corresponding Raman spectra also indicate the crystalline Raman peaks of the HfSe_2_ and WSe_2_ with fewer changes related to the position (Figure S8h, Supporting Information). Although only 2‐inch heterostructure wafers are fabricated due to equipment limitations, the proposed method can be scaled to produce larger wafers while maintaining high‐quality and clean interfaces.

### 2.4 Interlayer Coupling and Charge Transfer in the Heterostructures

**Figure 4:**
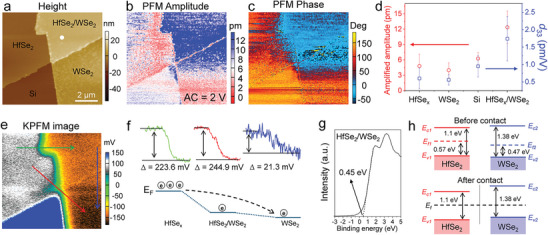
Investigation of interlayer coupling and charge transfer of HfSe_2_/WSe_2_ heterostructure. a–c) Height image, PFM amplitude image, and PFM phase image of HfSe_2_, WSe_2_, and HfSe_2_/WSe_2_ heterostructure. d) Single frequency PFM amplitude and calculated *d*_33_ of HfSe_2_, Si, WSe_2_, and HfSe_2_/WSe_2_ heterostructure. e) KPFM image of HfSe_2_/WSe_2_ heterostructure. f) Determined relative positions of Fermi level of HfSe_2_, WSe_2_, and HfSe_2_/WSe_2_ heterostructure. g) XPS valence band edge of HfSe_2_/WSe_2_ heterostructure. h) Band alignment of HfSe_2_/WSe_2_ heterostructure before and after contact.

Interlayer coupling is indispensable for engineering band energy alignment and ensuring highly efficient charge transfer in heterostructures. Thus, the interfacial coupling of HfSe_2_/WSe_2_ interlayer heterostructures prepared by interface‐enhanced selenization is investigated through out‐of‐plane piezoelectric force microscopy (PFM) and Kelvin probe force microscopy (KPFM), aimed at exploring the electric‐induced polarization and Fermi carrier transfer (**Figure** **4**). As illustrated in Figure 4a–c, the piezoelectric signals in the HfSe_2_/WSe_2_ heterostructure are noticeably stronger than in individual HfSe_2_, WSe_2_, and Si substrates. The resonance curves collected from a white spot in Figure 4a show resonance peaks at 244 kHz (Figure S9a, Supporting Information), corresponding to out‐of‐plane piezoelectricity. The peak intensity rises with an increase in the applied alternating current (AC) voltage, confirming the piezoelectric behavior of the HfSe_2_/WSe_2_ heterostructure (Figure S9a, Supporting Information). The emergence of piezoelectricity in the heterostructure is reasonable as the top and bottom vdW layers induce asymmetric polarization under external mechanical strain. The intrinsic amplitude and phase images are derived by fitting the amplitude and phase images using a simple harmonic oscillator (Figure S9b,c, Supporting Information). Despite some missing pixels, the actual out‐of‐plane piezoelectric coefficient (*d*_33_) can be estimated by dividing the statistical amplitudes by the applied voltage (2 V). The *d*_33_ values of HfSe_2_, Si, and WSe_2_ are smaller than 1 pm V^−1^, and constant phase degrees are observed in the fitted phase images, assigned to impurities or the changes of the surface morphology (Figure 4d; Figure S9b,c, Supporting Information). However, the *d*_33_ of the HfSe_2_/WSe_2_ heterostructure is 1.74 pm V^−1^ (Figure 4d), assigned to the inverse piezoelectric effect of the heterostructure, as indicated by the distinct phase shift and strong amplitude response.

The KPFM analysis reveals different Fermi levels across regions, with HfSe_2_ showing the highest KPFM response and WSe_2_ the lowest (Figure 4e,f). The work function difference between the pure HfSe_2_ and WSe_2_ layers is ≈244.9 mV, while the Fermi level of the HfSe_2_/WSe_2_ heterostructure is 223.6 mV lower than that of HfSe_2_ and 21.3 mV higher than that of WSe_2_. The change in work function within the HfSe_2_/WSe_2_ heterostructure indicates effective electron transfer from HfSe_2_ to WSe_2_, or hole transfer from WSe_2_ to HfSe_2_, suggesting a high‐quality interface in the heterostructure (Figure 4f). Band diagrams of HfSe_2_ and WSe_2_ before contact are constructed by referring to the prior studies on HfSe_2_ and WSe_2_ band energy levels and the measured XPS valence band edge of the HfSe_2_/WSe_2_ heterostructure, showing a type‐I straddling band alignment (Figure 4g,h).^[^ ^39^, ^40^ ^]^ The determined Fermi level of HfSe₂ is ≈0.57 eV above the valence band maximum, consistent with the value observed for HfSe₂ epitaxially grown on HOPG substrates by molecular beam epitaxy.^[^ ^39^ ^]^ However, as the Fermi level difference between HfSe_2_ and WSe_2_ overcomes the difference of *E*_v_ valence band maximum, the band alignment shifts to a typical type‐II semiconductor heterojunction upon contact (Figure 4h). The depletion layer and internal electric field formed at the heterostructure interface allow carriers to pass in a single direction, potentially creating a rectifier diode.

**Figure 5:**
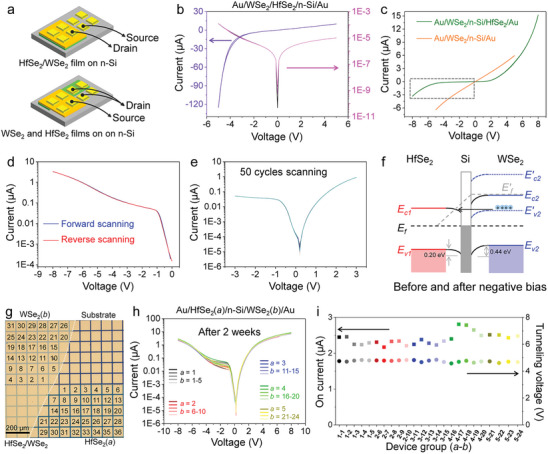
Electrical behaviors of WSe_2_/HfSe_2_ heterostructures and 2D film‐Si integrated heterojunctions. a) Schematic illustration of the two‐terminal device with Au/WSe_2_/HfSe_2_/n‐Si/Au and Au/WSe_2_/n‐Si/HfSe_2_/Au junctions. b) *I*–*V* curves of Au/WSe_2_/HfSe_2_/n‐Si/Au junctions with linear or log scale. c) *I*–*V* curves of Au/WSe_2_/n‐Si/HfSe_2_/Au and Au/WSe_2_/n‐Si/Au junctions. d) Forward and reverse scanning of *I–V* curves in log scale, as marked in the dashed line box in (c). e) Cycling and endurance tests of Au/WSe_2_/n‐Si/HfSe_2_/Au junctions. f) Schematic of band alignment of Au/WSe_2_/n‐Si/HfSe_2_/Au before and after applying negative bias. g) Schematic and optical images of the concept of the device group combination. h) *I*–*V* curves of 24 device groups after two weeks of storage in ambient air. i) Correspondingly recorded on‐currents and tunneling voltages of 24 device groups.

To explore the electrical characteristics of the HfSe_2_/WSe_2_ interlayer heterostructure, two‐terminal devices are fabricated on an n‐doped Si substrate using a rigid mask, followed by electrode deposition. Two types of device structures are manufactured: Au/WSe_2_/HfSe_2_/n‐Si/Au and Au/WSe_2_/n‐Si/HfSe_2_/Au junctions (**Figure** **5**a). Owing to the lower work function of n‐doped Si and the proximity of the Fermi level of HfSe₂ to the conduction band edge (*E*_c_), an Ohmic contact forms between n‐Si and HfSe₂, with n‐Si/Au serving as the electrode. *I*–*V* curves of the Au/WSe_2_/HfSe_2_/n‐Si/Au heterojunction show an obvious rectification effect, with a rectification ratio of 12.5 (Figure 5b), which aligns well with the band alignment analysis of the heterostructure. Given the low barrier height at the interface of the heterostructure, the rectification ratio is not significantly high. However, compared with stacked WSe_2_/HfSe_2_ heterostructure fabricated via mechanical exfoliation of WSe_2_ and HfSe_2_ crystals, the rectification effect of HfSe_2_/WSe_2_ interlayer heterostructure grown via one‐pot interface‐enhanced selenization is more obvious (Figure S10, Supporting Information). This confirms the successful HfSe_2_/WSe_2_ heterostructure preparation through one‐pot interface‐enhanced selenization. Moreover, scanning in opposite directions reveals a negligible hysteresis, indicating low trap defects at the interface of the HfSe_2_/WSe_2_ interlayer heterostructure (Figure 5b).

As the 2D films are directly prepared on the Si substrate, the heterogeneous integration of 3D Si and 2D films can be realized to supplement Si‐based integrated circuits. Both the Au/WSe_2_/n‐Si/Au and Au/WSe_2_/n‐Si/HfSe_2_/Au junctions demonstrate 3D‐2D heterostructures. The Au/WSe_2_/n‐Si/Au junction shows typical linear behavior, confirming Ohmic contact between the 2D WSe_2_ layer and n‐Si (Figure 5c). The Au/WSe_2_/n‐Si/HfSe_2_/Au junction displays similar rectification behavior to the HfSe_2_/WSe_2_ heterostructure (Figure 5c), but experiences breakdown at the cutoff region when the voltage exceeds 4 V (Figure 5d). Forward and backward voltage scans show excellent overlap of the curves even after 50 cycles (Figure 5e), suggesting the high‐quality interface and durable stability of the 3D‐2D heterostructure. The band alignment analysis suggests that the breakdown is a Zener breakdown. As the negative voltage increases, the energy band of WSe_2_ rises, allowing electrons in the valence band of WSe_2_ to tunnel through the band barrier to the conduction band of HfSe_2_, resulting in tunneling current breakdown of the diodes (Figure 5f). These Zener tunneling behaviors suggest that 2D films and heterostructures fabricated using one‐pot interface‐enhanced selenization may produce tunnel diodes, which could be used in switch circuits or high‐frequency oscillators.

To verify the environmental stability and performance repeatability of the Au/WSe₂/n‐Si/HfSe₂/Au junction, the electrical characteristics of 24 device groups, each with different WSe₂ and HfSe₂ pattern combinations, are measured after two weeks of exposure to ambient air. The concept of device group combination is illustrated in Figure 5g, and the actual devices are shown in Figure S11a (Supporting Information). Each Au pattern measures ≈100 µm × 100 µm, and the gap between each Au pad is 10 µm. The *I*–*V* curves of the 24 device groups exhibit similar trends, showing minimal variation in on‐current and tunneling voltage (Figure 5h,i; Figure S11b, Supporting Information), confirming the homogeneous crystallization of the WSe_2_ and HfSe_2_ films. Notably, the *I–V* curve shape of the devices exposed to ambient air for 2 weeks and 5 months remains comparable to that of pristine devices (Figure 5e,h; Figure S12a, Supporting Information). However, the off‐current decreases from the pristine value of 3.3 to 1.4 µA after 5 months of ambient air exposure. By fitting the currents to time using an exponential damping function, the results demonstrate reliable long‐term stability over 1 year under ambient air conditions. Considering that transistors typically operate in encapsulated environments, these findings suggest that the devices exhibit substantial long‐term reliability. Additionally, the devices were annealed in air to assess the thermal stability of the heterostructure. As the annealing temperature increases, the tunnelling breakdown behaviour of the diode gradually weakens and eventually disappears after annealing at 200 °C in the air (Figure S12c–f, Supporting Information). After annealing at 250 °C for 10 min, the *I–V* curve becomes rough and unstable (Figure S12g, Supporting Information), indicating that the surface and interface of the heterostructure have been damaged. These results suggest the excellent resistance of the 3D‐2D heterostructure devices to ambient air and thermal degradation, highlighting the potential rectification function of the 3D‐2D heterostructure and its suitability for integration with Si‐compatible transistors.

## 3 Conclusion and Perspectives

In summary, we successfully achieved the selenization of low‐reactivity metallic Hf films and guided the growth of HfSe_2_/WSe₂ interlayer heterostructures, characterized by a high‐quality interface, suitable band alignment, and excellent stability. The proposed method broadens the potential candidates for fabricating 2D heterostructures and holds promise for wafer‐scale fabrication. Notably, the vdW planes of the interlayer heterostructure, aligned perpendicular to the substrate, significantly enhance surface area utilization, increase device intensity, and ensure compatibility with Si wafers for transistor integration. Especially, magnetron sputtering facilitates the preparation of a wide range of metal films with excellent uniformity and densification on various substrates. Furthermore, metals such as W, Mo, V, Nb, and Pt exhibit strong reactivity with chalcogens, making the one‐pot method highly promising for the fabrication of other 2D metal chalcogenide heterostructures. However, challenges remain, particularly regarding the incomplete selenization of Hf, necessitating precise control and optimization of Hf film thickness, selenization temperature, and selenization duration. Additionally, preventing alloy formation is crucial for adapting the one‐pot method to the preparation of other heterostructures. Metals with varying reactivities toward chalcogens generally contribute positively to the fabrication of 2D heterostructures with well‐defined interfaces. This work provides novel insights into preparing interlayer heterostructures using low‐sulfidation‐activity metals, potentially contributing to the advancement of wafer‐scale fabrication and technological applications of 2D material heterostructures.

## 4 Experimental Section

### Preparation of 2D Interlayer Heterostructures

The standard DC magnetron sputtering process for the preparation of metal films involved silicon wafers or silicon wafers with oxide layers as substrates and 2‐inch Hf or W targets (purity: 99.995%) as sources. During deposition, the pressure of the sputtering chamber was maintained at 3 × 10^−3^ Torr, tuned by a constant flow of 30 sccm Ar gas. For W and Hf deposition, the sputtering power was set as 40 and 20 W, respectively. After 10 min deposition, the sputtering power was turned off, and the sample was stored in a vacuum environment in preparation for selenization.

Selenization of metal films was performed in a single‐zone tube furnace. First, the tube furnace was annealed in an Ar and H_2_ atmosphere at 500 °C to remove residual oxygen. Subsequently, the metal film was placed at the center of the tube furnace, and the Se particles were placed in the upstream zone. The temperature was raised to the target temperature over 1 h, with Ar and H_2_ gas flows maintained at 200 sccm. After 1 h of isothermal selenization, the tube furnace was gradually cooled to room temperature (25 °C).

The distinction between the stepwise and one‐pot preparation methods lies in the sequential versus one‐time selenization of metal films. In the stepwise method, the Hf film was selenized first, followed by W film deposition and subsequent selenization (Figure 1a). In contrast, in the one‐pot preparation method, the Hf/W metal films deposited by magnetron sputtering were directly selenized (Figure 1b). The temperature and airflow of the two methods were identical.

### Characterizations and Device Fabrications

AFM, KPFM, and PFM characterizations were performed using Cypher S instruments at room temperature under ambient air. Silicon tips with a force constant of 2.8 N/m and Pt/lr conductive coating were used for KPFM and PFM characterizations. XPS spectra were obtained using a Thermo Fisher Scientific Nexsa G2 X‐ray photoelectron spectrometer. Scanning electron microscopy and EDS characterizations were performed using a field‐emission scanning electron microscope (FEI, Nano SEM 450). Raman spectra were recorded using a confocal Raman spectrometer with a laser source of 532 nm (Witec alpha300 R). TEM characterizations were performed using an FEI Talos F200S instrument after 50 nm carbon deposition and FIB cutting.

A physical masking method was used to fabricate the two‐terminal devices of the heterostructure. The channel was first sheltered by Cu grids with 10 µm channels and 120 µm blank windows. Subsequently, Ti/Au (5/50 nm) electrodes were deposited using a Denton E‐beam deposition system. The electrical measurements were conducted in a probe station with a Keysight B2902B parameter analyzer at room temperature and atmospheric pressure.

## Conflict of Interest

The authors declare no conflict of interest.

## Author Contributions

Y.H. and X.W. contributed equally to this work. Y.H., X.W., S.P.L., and B.K.T. conceived this work. Y.H. and X.W. prepared materials. Y.H., X.W. and Y.G. performed AFM characterizations and analysis. Y.H., Z.T., X.W., XPS, and XRD performed characterizations. Y.H. and X.W. performed Raman characterizations and analysis. Y.H., J.L. and W.H.Y. performed EDS, TEM and STEM characterizations and analysis. Y.H., Y.G., X.W., G.Z., W.H.Y. and S.B.L. fabricated devices and performed electrical measurements. Y.H. wrote the manuscript with input from X.W., M.B., P.C., S.P.L. and B.K.T. All authors discussed the results and commented on the manuscript. M.B., P.C., S.P.L. and B.K.T. supervised the project.

## Supporting information



Supporting Information

## Data Availability

The data that support the findings of this study are available from the corresponding author upon reasonable request.
