# Supplementary material for: Interface‐Enhanced and Self‐Guided Growth of 2D Interlayer Heterostructure Wafers with Vertically Aligned Van Der Waals Layers
Source: Adv Sci (Weinh). 2025 Feb 17;12(14):2412690. doi: 10.1002/advs.202412690 (PMC11984839; doi:10.1002/advs.202412690)
Supplement: Supplementary file 1 — Supporting Information [file ADVS-12-2412690-s001.docx]

**Supporting Information for:**

**Interface-enhanced and Self-guided Growth of 2D Interlayer Heterostructure Wafers with Vertically Aligned Van Der Waals Layers**

*Yi Hu,^1,3#^ Xingli Wang,^1,2#^ Xingguo Wang,^1,2^ Yue Gong,^1,4^ Zikun Tang,^3^ Guangchao Zhao,^1,2^ Weng Hou Yip,^1,2^ Jingyi Liu, ^1^ Seoung Bum Lim, ^1^ Mohamed Boutchich, ^2,5^ Philippe Coquet,^2,6^ Shu Ping Lau,^3^* Beng Kang Tay,^1,2,^**

**Affiliations:**

^1^ Centre for Micro- and Nano-Electronics (CMNE), School of Electrical and Electronic Engineering, Nanyang Technological University, Singapore 638798, Singapore.

^2^ CINTRA IRL 3288 (CNRS NTU THALES), Nanyang Technological University, Singapore 637553.

^3^ Department of Applied Physics, Hong Kong Polytechnic University, Hung Hom, Kowloon, Hong Kong, P. R. China.

^4^ Interdisciplinary Graduate School, Nanyang Technological University, Singapore 639798, Singapore.

^5^ Sorbonne Université, CNRS, Laboratoire de Génie Electrique et Electronique de Paris, 75252 Paris, France.

^6^ Univ. Lille, CNRS, Institut d’Electronique, Microélectronique et Nanotechnologie, IEMN, France 60069.

* Corresponding author. Email: apsplau@polyu.edu.hk (S.P.L.), ebktay@ntu.edu.sg (B.K.T.).

# Contributed equally.


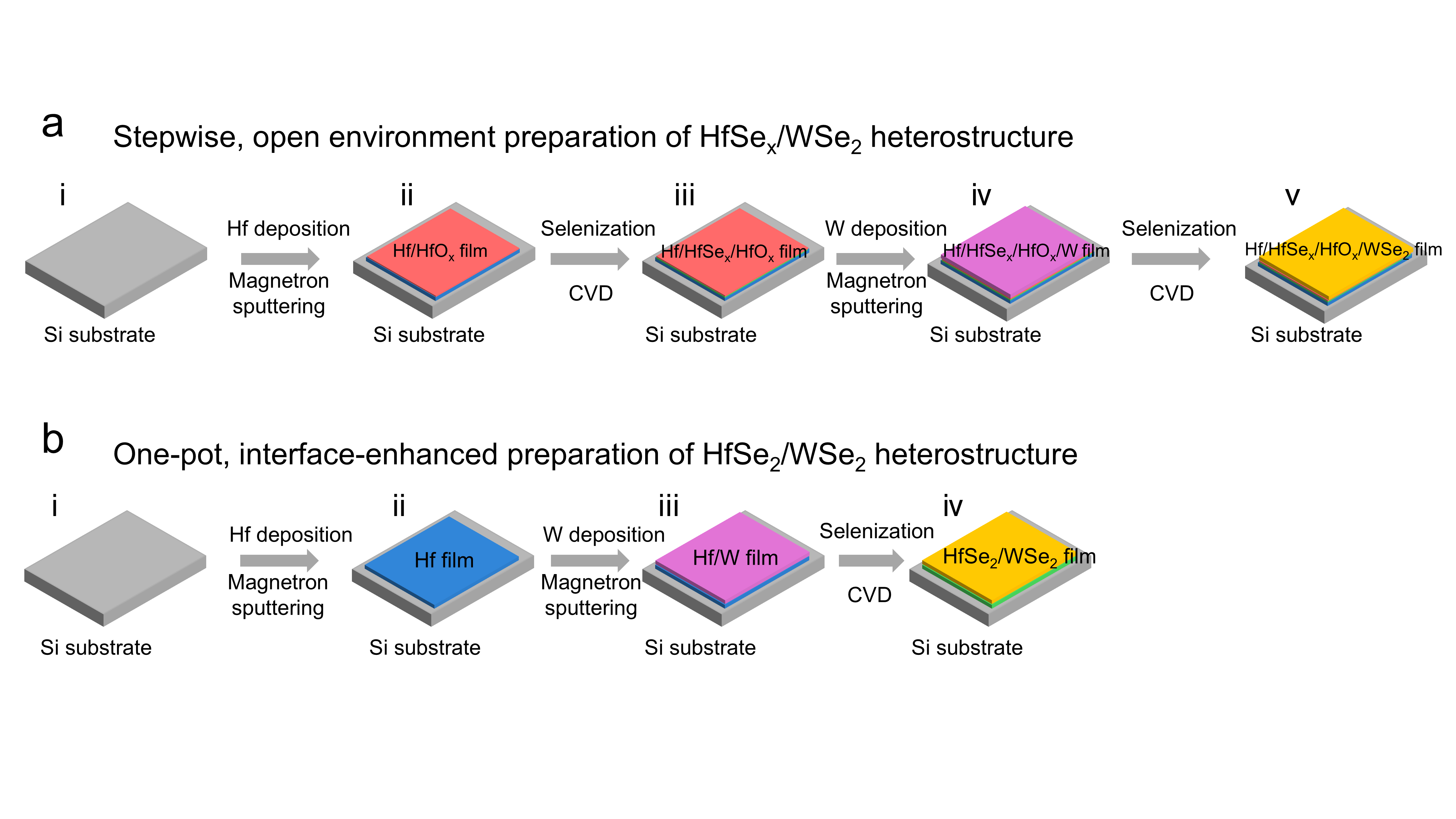


**Figure S1.** (a) Schematic illustration of step-by-step metal film deposition and selenization procedure. (b) Schematic illustration of one-pot selenization of Hf/W metal films with interface-enhanced selenization.


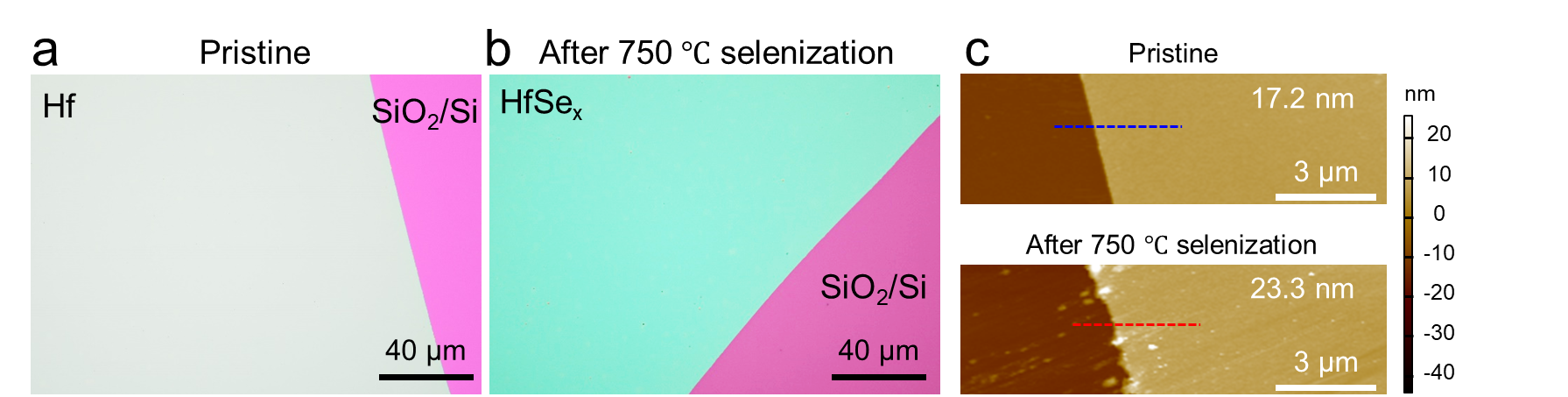


**Figure S2.** (a) Optical image of as-deposited Hf metal film on SiO_2_/Si substrate. (b) Optical image of Hf metal film on SiO_2_/Si substrate after selenization at 750 ℃. (c) Atomic force microscopy (AFM) images of pristine Hf film (top panel) and the film after 750 ℃ selenization (bottom panel).


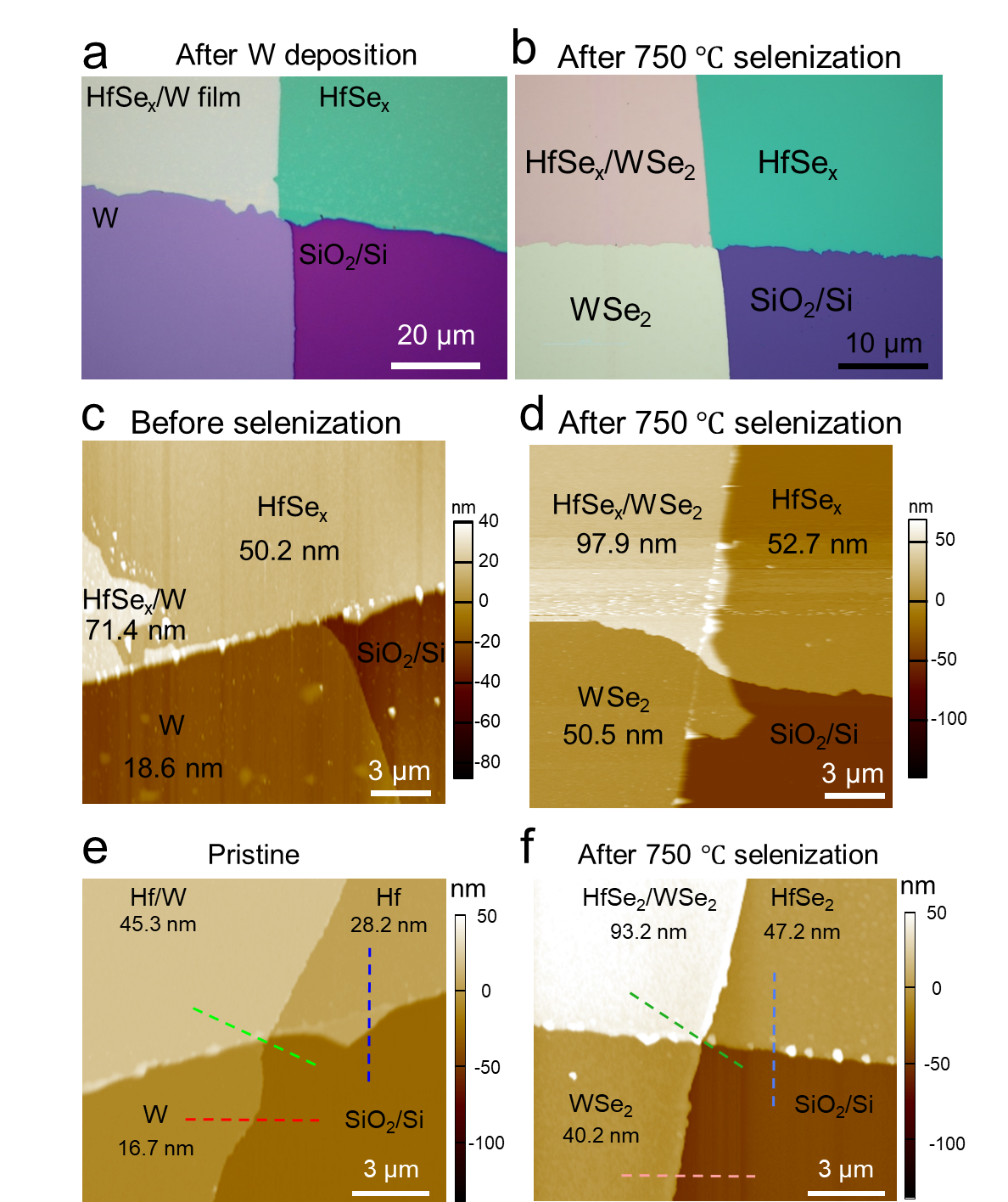


**Figure S3.** (a) Optical image of HfSe_x_/W hetero-films on SiO_2_/Si substrate. (b) Optical image of HfSe_x_/W hetero-films on SiO_2_/Si substrate after selenization at 750 ℃. (c-d) Corresponding AFM images of HfSe_x_/W hetero-films on SiO_2_/Si substrate (c) before and (d) after 750 ℃ selenization.


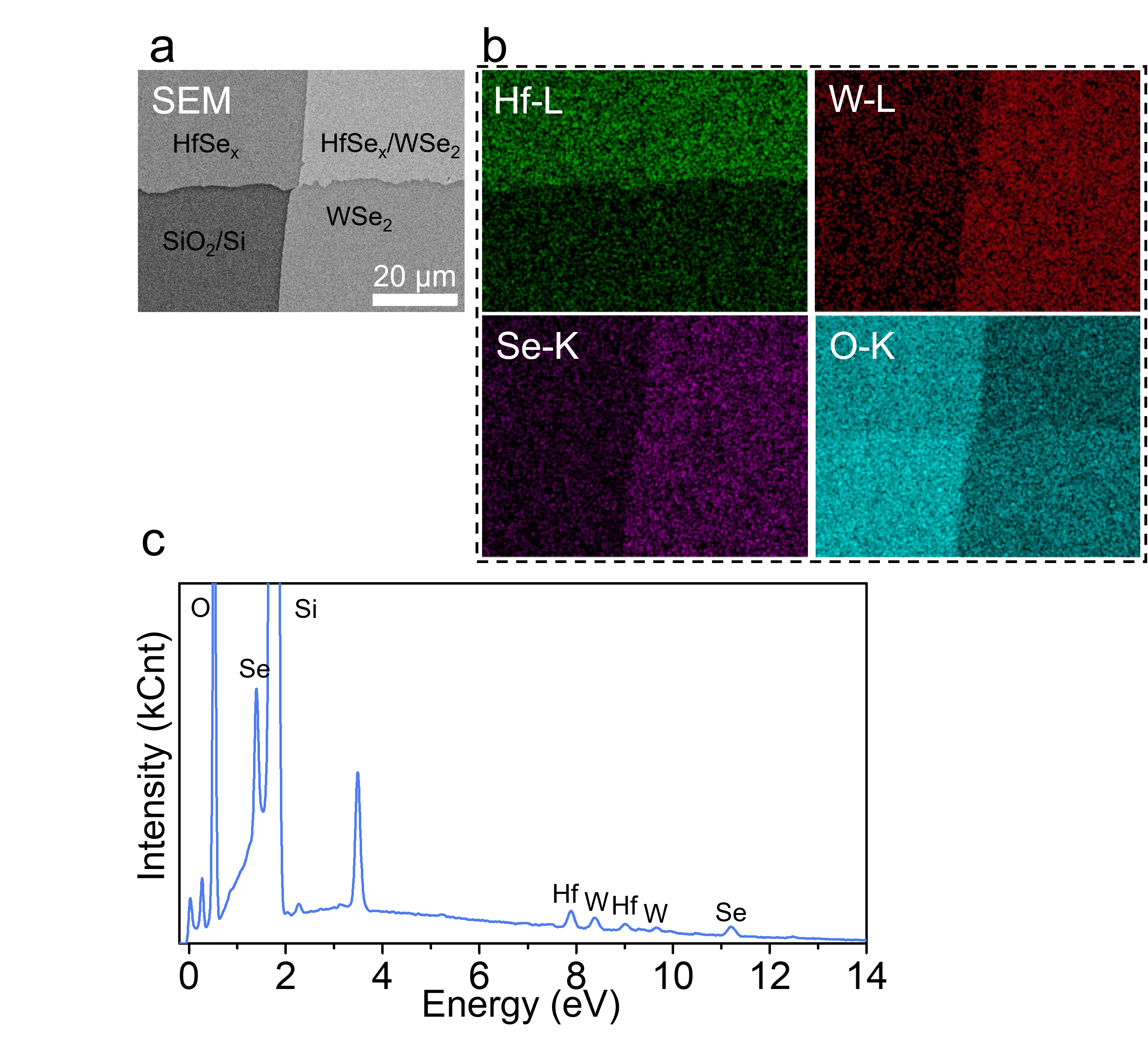


**Figure S4.** (a,b) SEM image and corresponding elemental mapping images of Hf, W, Se, and O of HfSe_x_/WSe_2_ heterostructure prepared by stepwise metal deposition and selenization. (c) Corresponding EDS spectrum of HfSe_x_/WSe_2_ heterostructure.

**
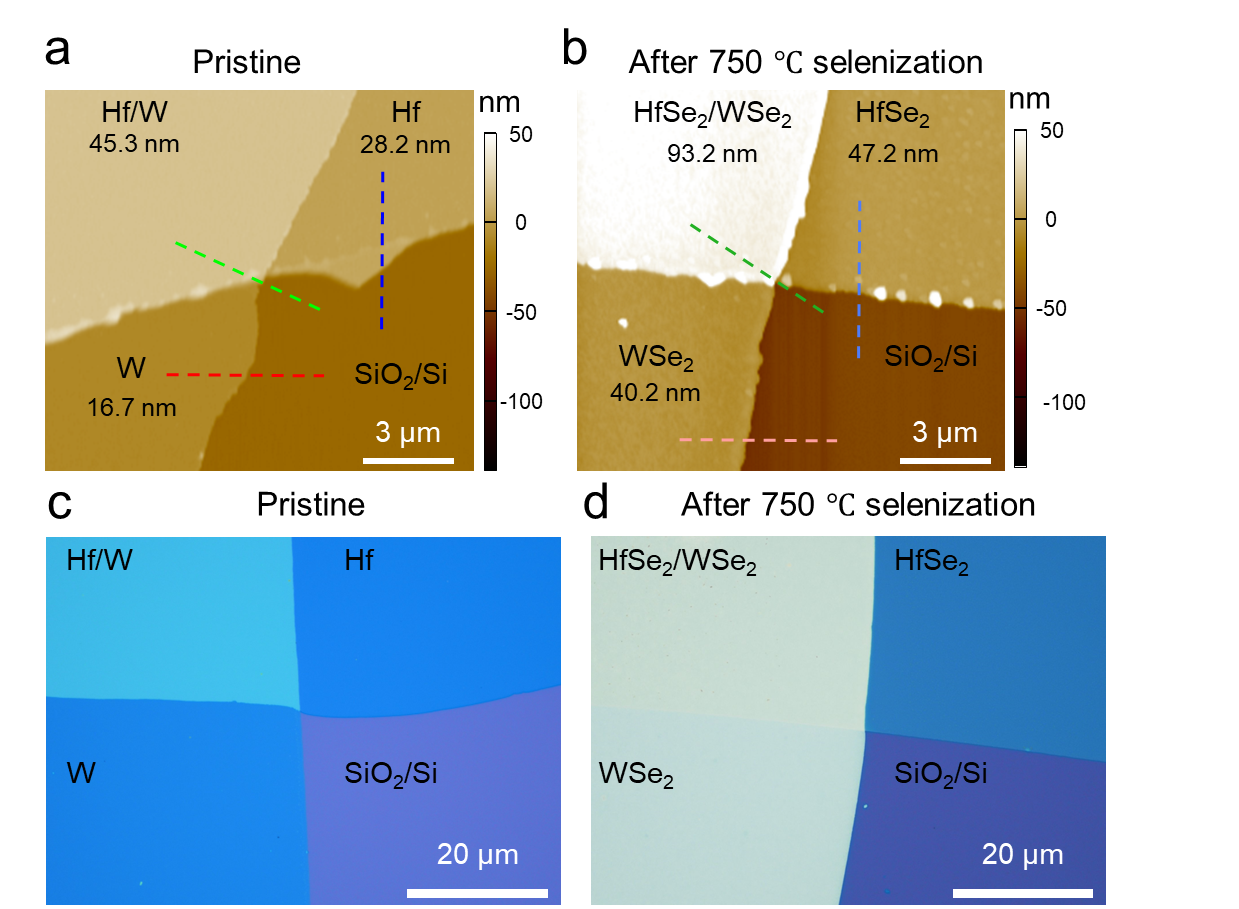
**

**Figure S5.** (a–b) AFM images of Hf/W, W, and Hf films (a) before and (b) after one-pot interface-enhanced selenization at 750 ℃.(c-d) Optical images of Hf/W films (c) before and (d) after 750 ℃selenization.


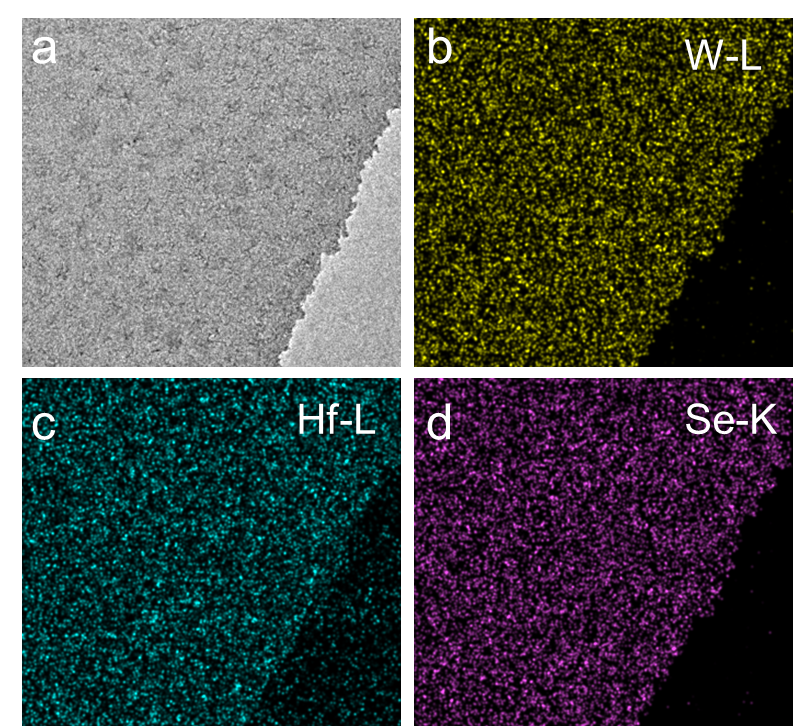


**Figure S6**. (a-d) The TEM image and corresponding W, Hf and Se element mapping images of the HfSe_2_/WSe_2_ heterostructure collected from the top-view.


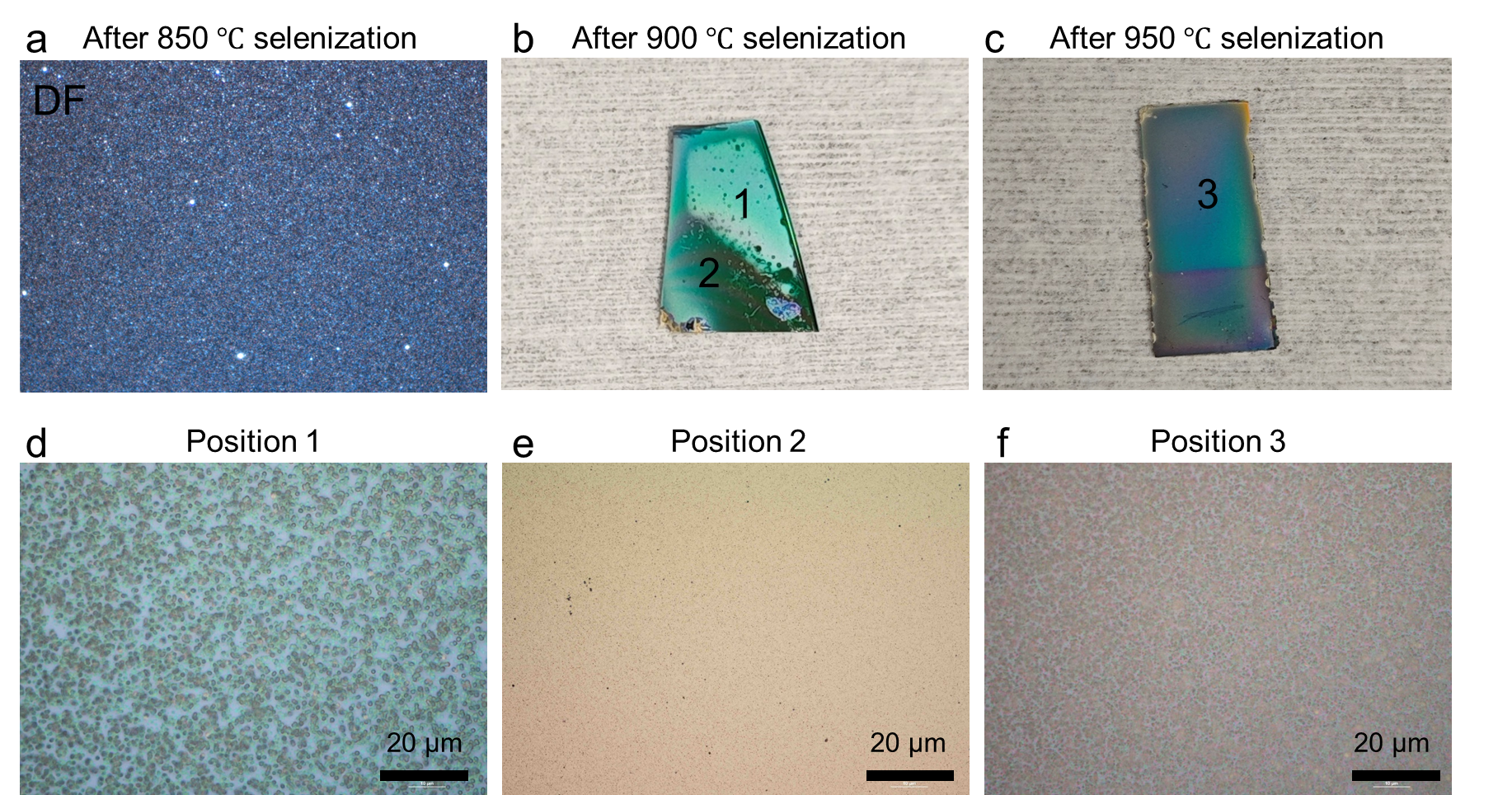


**Figure S7.** (a) DF optical image of the Hf/W films after selenization at 850 ℃. (b-c) Digital images of Hf/W metal films after selenization at (b) 900 ℃ and (c) 950 ℃, respectively. (d-f) Optical images collected from three positions marked in (b) and (c).

**
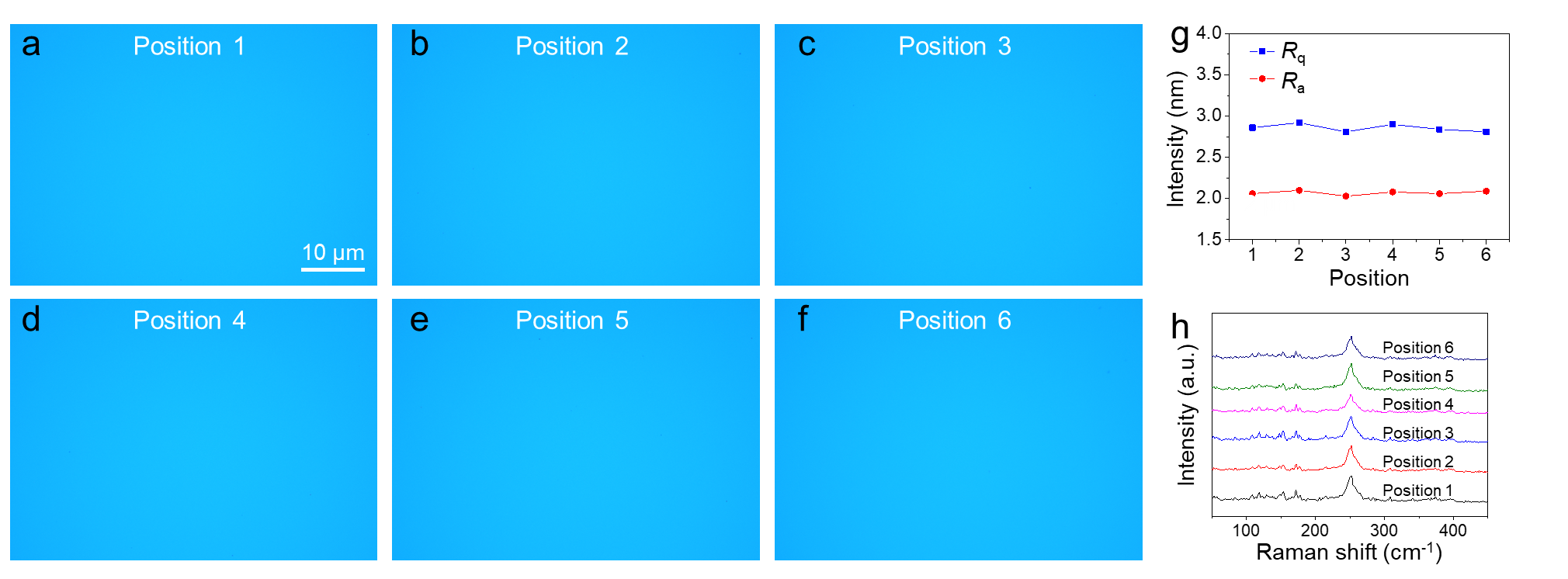
Figure S8.** (a-f) Optical images collected on six points of the heterostructure wafer in Figure 3h. (g) Surface roughness (*R*_a_ and *R*_q_) parameters of six points on the 2-inch HfSe_2_/WSe_2_ heterostructure wafer. (h) Corresponding Raman spectra of six points on the 2-inch HfSe_2_/WSe_2_ heterostructure wafer.


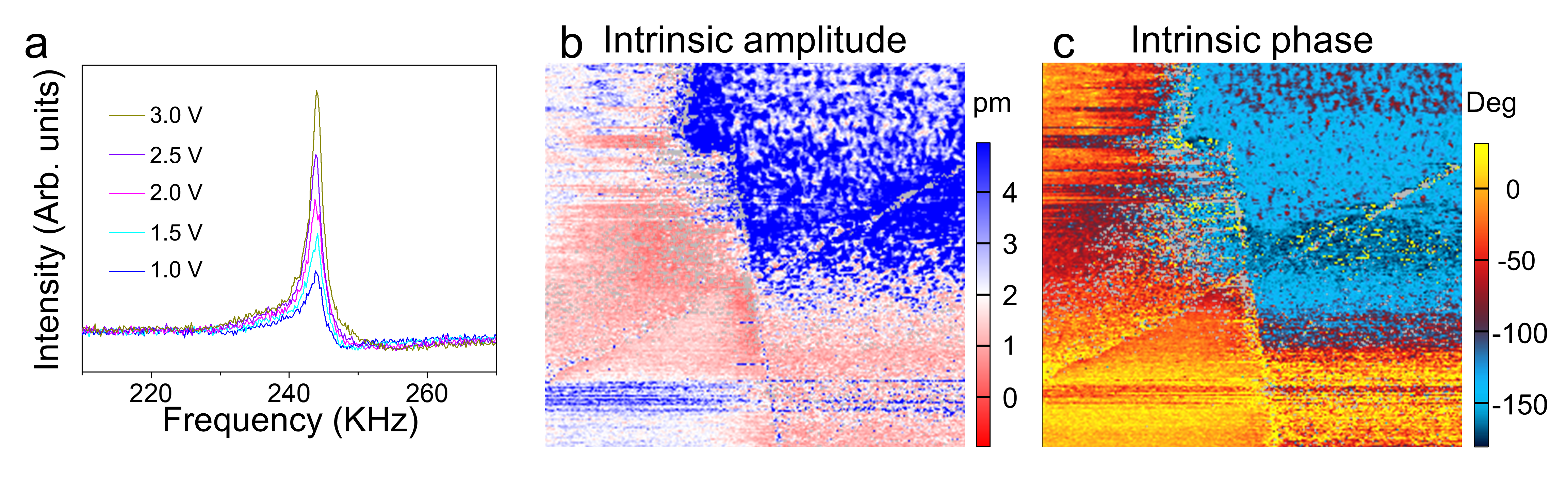


**Figure S9.** (a) The voltage-dependent resonance curves of HfSe_2_/WSe_2_ heterostructure prepared by interface-enhanced selenization. (b-c) Corresponding intrinsic PFM amplitude and phase images of HfSe_2_/WSe_2_ heterostructure after a simple harmonic oscillator (SHO) fitting.


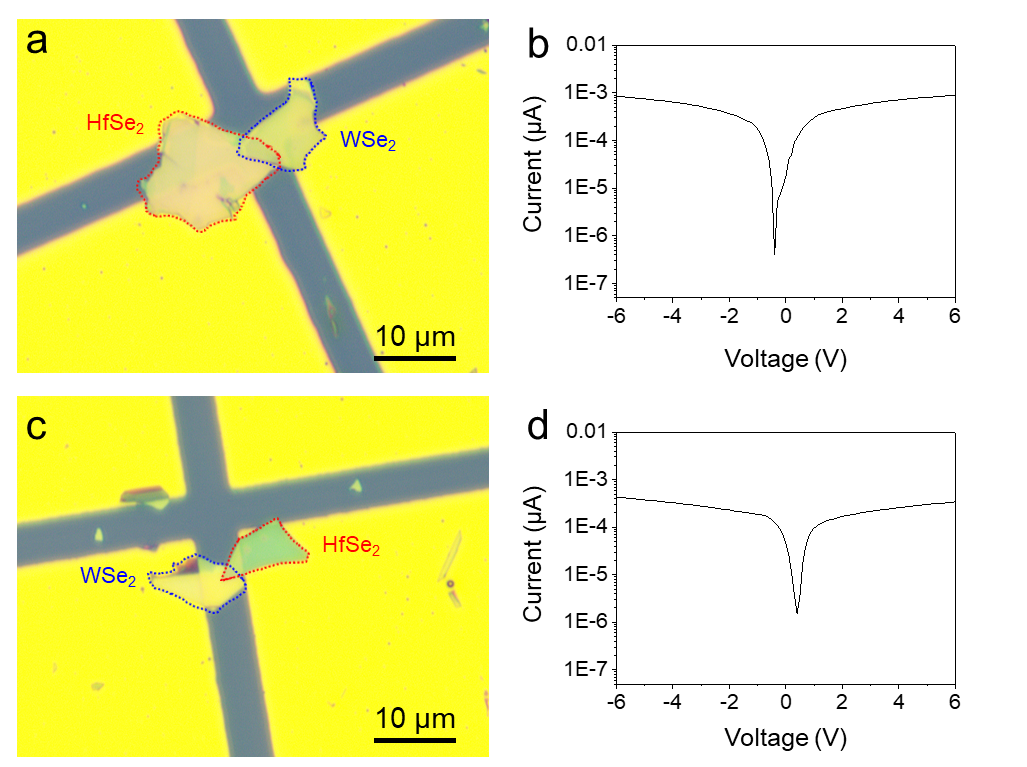


**Figure S10.** (a) Optical image of a WSe_2_/HfSe_2_ interlayer heterostructure fabricated via mechanical exfoliation and stacking. The WSe_2_ layer is on the top of the HfSe_2_ layer. (b) Corresponding *I*-*V* curve with a log scale. (c) Optical image of a HfSe_2_/WSe_2_ interlayer heterostructure stacked via mechanically exfoliated WSe_2_ and HfSe_2_ layers. The HfSe_2_ is on the top of the WSe_2_ layer. (d) Corresponding *I*-*V* curve with log scale of the device in (c).


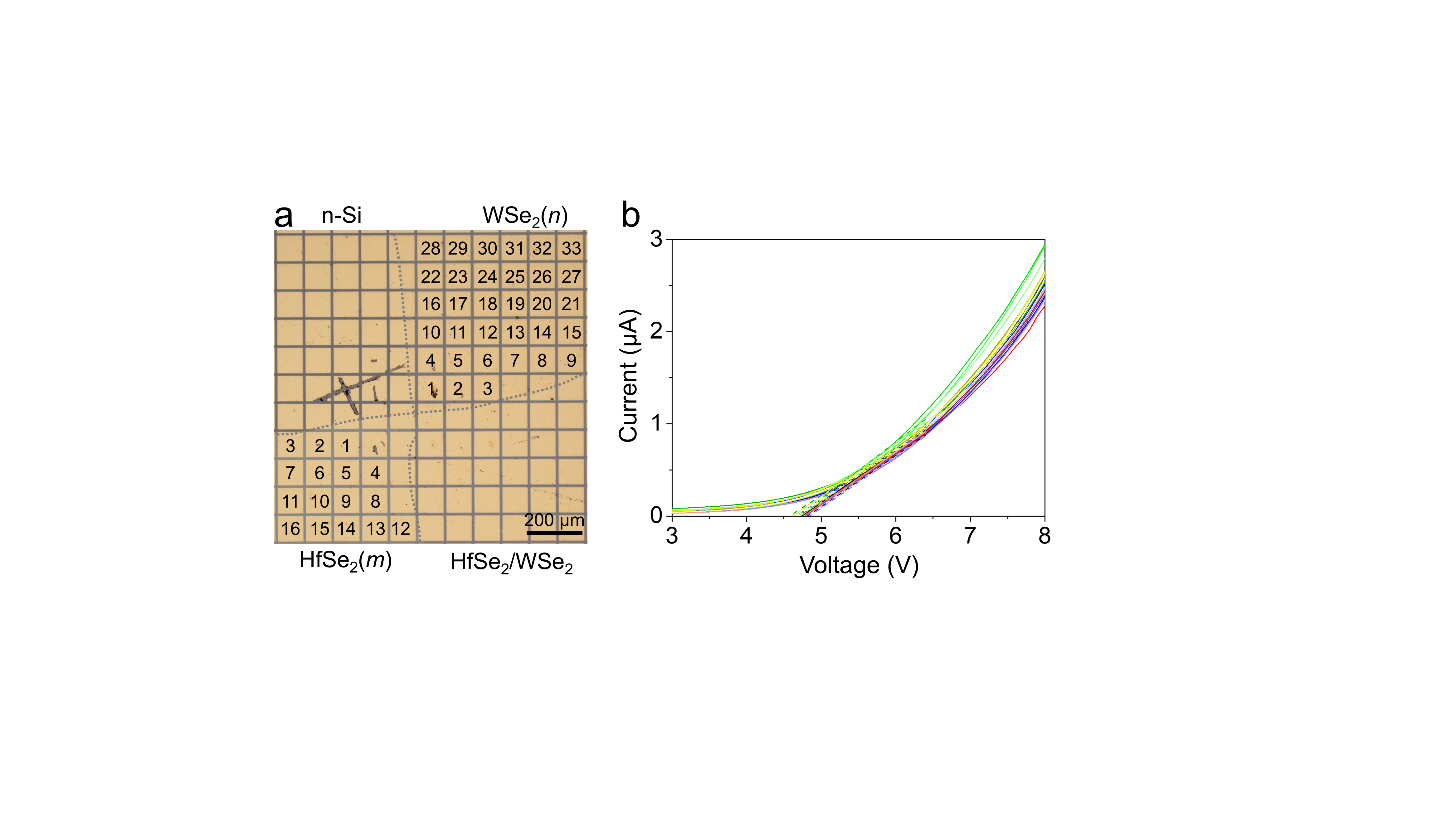


**Figure S11.** (a) Optical image of Au electrode-patterned HfSe_2_/WSe_2_ heterostructure on Si substrate. (b) Magnified *I-V* curves of 24 HfSe_2_(*a*)/n-Si/WSe_2_(*b*) devices. The tunnel breakdown voltages were estimated using linear fitting.


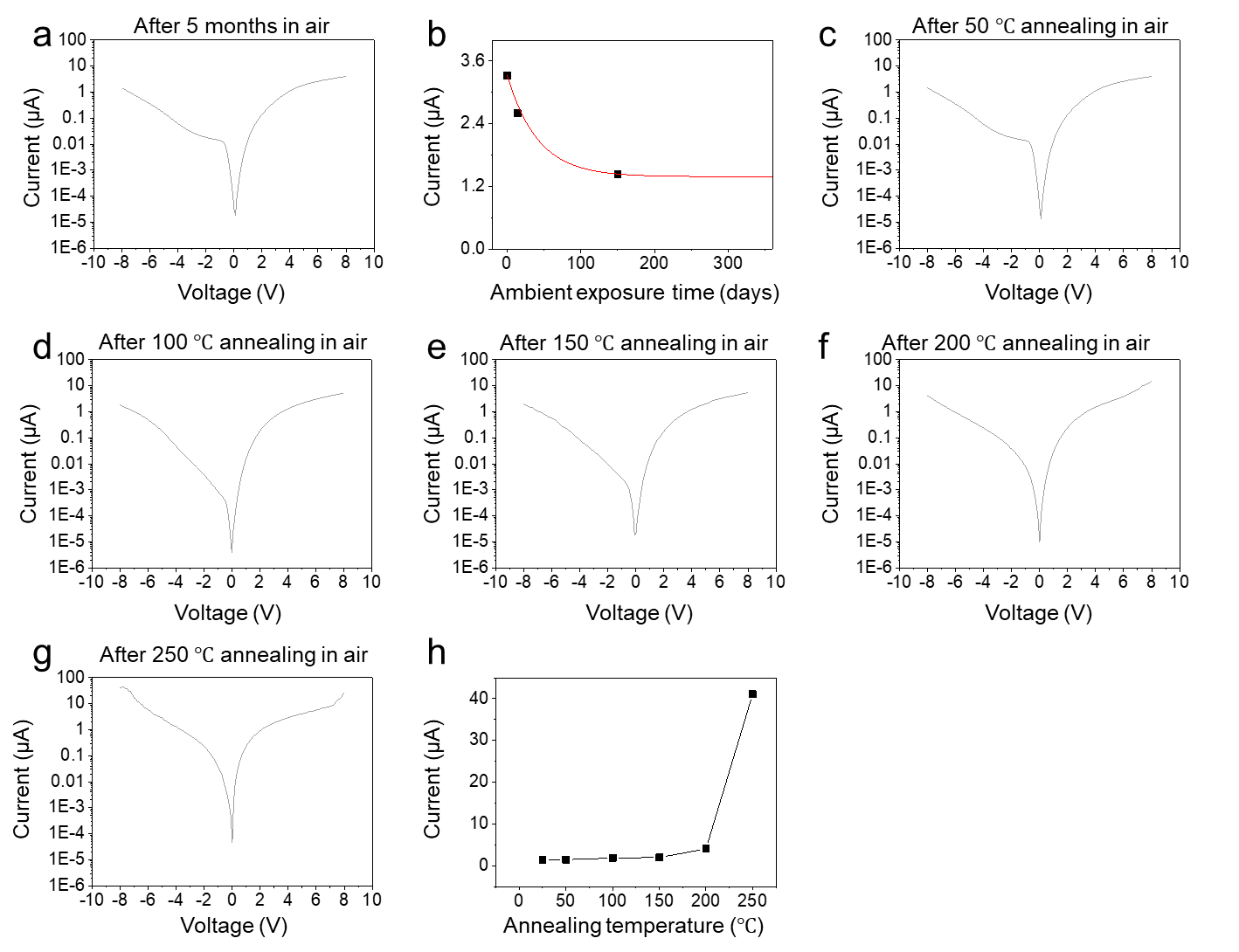


**Figure S12.** (a) *I-V* curve of an Au/WSe_2_/n-Si/HfSe_2_/Au junction after exposure to ambient air for 5 months. (b) Corresponding current decay in ambient air with respect to time. (c-g) *I-V* curves of an Au/WSe_2_/n-Si/HfSe_2_/Au junction after annealing in ambient air for 10 mins, the annealing temperatures are set at (c) 50 ℃, (d) 100 ℃, (e) 150 ℃, (f) 200 ℃ and (g) 250 ℃, respectively. (h) Corresponding current variations related to annealing temperature.
